# Supplementary material for: Allosteric regulation and crystallographic fragment screening of SARS-CoV-2 NSP15 endoribonuclease
Source: Nucleic Acids Res. 2023 Apr 28;51(10):5255–70. doi: 10.1093/nar/gkad314 (PMC10250223; doi:10.1093/nar/gkad314)
Supplement: gkad314_Supplemental_Files [file gkad314_supplemental_files.zip › Supplementary datasets.zip!/81/Refine_9/01-BUSTER/Cycle-5/shell.01/R_Rfree.html]

# Rxpct factors in resolution bins at cycle 0


|  |  |  |  |  |  |  |  |  |
| --- | --- | --- | --- | --- | --- | --- | --- | --- |
|  | **<d> (Å)**  Rxpct\_Work | Rxpct\_Free | Rxpct\_All | |  |  |  |  |
|  | 15.0454 | 0.2803 | 0.2378 | 0.2787 |  |
|  | 8.6865 | 0.2152 | 0.2645 | 0.2181 |  |
|  | 6.7285 | 0.2534 | 0.2339 | 0.2522 |  |
|  | 5.6866 | 0.2653 | 0.2602 | 0.2651 |  |
|  | 5.0151 | 0.2093 | 0.2396 | 0.2112 |  |
|  | 4.5364 | 0.2005 | 0.2222 | 0.2016 |  |
|  | 4.1728 | 0.2138 | 0.1941 | 0.2128 |  |
|  | 3.8847 | 0.2325 | 0.2235 | 0.2321 |  |
|  | 3.6491 | 0.2375 | 0.2398 | 0.2376 |  |
|  | 3.4517 | 0.2475 | 0.2454 | 0.2474 |  |
|  | 3.2832 | 0.2465 | 0.2804 | 0.2486 |  |
|  | 3.1372 | 0.2385 | 0.2548 | 0.2393 |  |
|  | 3.0091 | 0.2368 | 0.2522 | 0.2374 |  |
|  | 2.8955 | 0.2242 | 0.2343 | 0.2247 |  |
|  | 2.7939 | 0.2164 | 0.2001 | 0.2157 |  |
|  | 2.7022 | 0.2016 | 0.2216 | 0.2026 |  |
|  | 2.6191 | 0.1880 | 0.2213 | 0.1899 |  |
|  | 2.5431 | 0.1863 | 0.1976 | 0.1870 |  |
|  | 2.4735 | 0.1809 | 0.1774 | 0.1807 |  |
|  | 2.4092 | 0.1905 | 0.1860 | 0.1903 |  |
|  | **Overall** | 0.2277 | 0.2339 | 0.2280 |  |

# Rxpct factors in resolution bins at cycle 1


|  |  |  |  |  |  |  |  |  |
| --- | --- | --- | --- | --- | --- | --- | --- | --- |
|  | **<d> (Å)**  Rxpct\_Work | Rxpct\_Free | Rxpct\_All | |  |  |  |  |
|  | 15.0454 | 0.2804 | 0.2379 | 0.2788 |  |
|  | 8.6865 | 0.2151 | 0.2646 | 0.2180 |  |
|  | 6.7285 | 0.2532 | 0.2341 | 0.2521 |  |
|  | 5.6866 | 0.2652 | 0.2603 | 0.2650 |  |
|  | 5.0151 | 0.2092 | 0.2397 | 0.2111 |  |
|  | 4.5364 | 0.2004 | 0.2222 | 0.2015 |  |
|  | 4.1728 | 0.2136 | 0.1940 | 0.2125 |  |
|  | 3.8847 | 0.2322 | 0.2234 | 0.2318 |  |
|  | 3.6491 | 0.2372 | 0.2398 | 0.2373 |  |
|  | 3.4517 | 0.2473 | 0.2453 | 0.2472 |  |
|  | 3.2832 | 0.2462 | 0.2803 | 0.2484 |  |
|  | 3.1372 | 0.2383 | 0.2547 | 0.2390 |  |
|  | 3.0091 | 0.2366 | 0.2522 | 0.2372 |  |
|  | 2.8955 | 0.2240 | 0.2342 | 0.2245 |  |
|  | 2.7939 | 0.2163 | 0.2000 | 0.2155 |  |
|  | 2.7022 | 0.2014 | 0.2217 | 0.2025 |  |
|  | 2.6191 | 0.1879 | 0.2213 | 0.1899 |  |
|  | 2.5431 | 0.1862 | 0.1977 | 0.1869 |  |
|  | 2.4735 | 0.1808 | 0.1774 | 0.1806 |  |
|  | 2.4092 | 0.1904 | 0.1861 | 0.1902 |  |
|  | **Overall** | 0.2275 | 0.2339 | 0.2278 |  |

# Rxpct factors in resolution bins at cycle 2


|  |  |  |  |  |  |  |  |  |
| --- | --- | --- | --- | --- | --- | --- | --- | --- |
|  | **<d> (Å)**  Rxpct\_Work | Rxpct\_Free | Rxpct\_All | |  |  |  |  |
|  | 15.0454 | 0.2804 | 0.2378 | 0.2788 |  |
|  | 8.6865 | 0.2149 | 0.2647 | 0.2178 |  |
|  | 6.7285 | 0.2527 | 0.2343 | 0.2516 |  |
|  | 5.6866 | 0.2646 | 0.2599 | 0.2644 |  |
|  | 5.0151 | 0.2088 | 0.2396 | 0.2107 |  |
|  | 4.5364 | 0.2001 | 0.2223 | 0.2012 |  |
|  | 4.1728 | 0.2132 | 0.1939 | 0.2121 |  |
|  | 3.8847 | 0.2318 | 0.2232 | 0.2314 |  |
|  | 3.6491 | 0.2368 | 0.2397 | 0.2369 |  |
|  | 3.4517 | 0.2469 | 0.2452 | 0.2468 |  |
|  | 3.2832 | 0.2459 | 0.2803 | 0.2481 |  |
|  | 3.1372 | 0.2379 | 0.2547 | 0.2387 |  |
|  | 3.0091 | 0.2364 | 0.2523 | 0.2370 |  |
|  | 2.8955 | 0.2239 | 0.2343 | 0.2244 |  |
|  | 2.7939 | 0.2162 | 0.1999 | 0.2154 |  |
|  | 2.7022 | 0.2013 | 0.2217 | 0.2024 |  |
|  | 2.6191 | 0.1879 | 0.2214 | 0.1899 |  |
|  | 2.5431 | 0.1861 | 0.1978 | 0.1868 |  |
|  | 2.4735 | 0.1809 | 0.1773 | 0.1807 |  |
|  | 2.4092 | 0.1904 | 0.1861 | 0.1902 |  |
|  | **Overall** | 0.2272 | 0.2338 | 0.2275 |  |

# Rxpct factors in resolution bins at cycle 3


|  |  |  |  |  |  |  |  |  |
| --- | --- | --- | --- | --- | --- | --- | --- | --- |
|  | **<d> (Å)**  Rxpct\_Work | Rxpct\_Free | Rxpct\_All | |  |  |  |  |
|  | 15.0454 | 0.2788 | 0.2335 | 0.2771 |  |
|  | 8.6865 | 0.2090 | 0.2635 | 0.2122 |  |
|  | 6.7285 | 0.2433 | 0.2383 | 0.2430 |  |
|  | 5.6866 | 0.2566 | 0.2542 | 0.2565 |  |
|  | 5.0151 | 0.2025 | 0.2331 | 0.2045 |  |
|  | 4.5364 | 0.1965 | 0.2201 | 0.1976 |  |
|  | 4.1728 | 0.2098 | 0.1946 | 0.2090 |  |
|  | 3.8847 | 0.2278 | 0.2240 | 0.2277 |  |
|  | 3.6491 | 0.2334 | 0.2397 | 0.2337 |  |
|  | 3.4517 | 0.2442 | 0.2413 | 0.2441 |  |
|  | 3.2832 | 0.2431 | 0.2794 | 0.2455 |  |
|  | 3.1372 | 0.2361 | 0.2581 | 0.2371 |  |
|  | 3.0091 | 0.2355 | 0.2532 | 0.2362 |  |
|  | 2.8955 | 0.2233 | 0.2363 | 0.2240 |  |
|  | 2.7939 | 0.2151 | 0.2010 | 0.2144 |  |
|  | 2.7022 | 0.2013 | 0.2274 | 0.2027 |  |
|  | 2.6191 | 0.1877 | 0.2234 | 0.1898 |  |
|  | 2.5431 | 0.1859 | 0.1990 | 0.1867 |  |
|  | 2.4735 | 0.1815 | 0.1784 | 0.1813 |  |
|  | 2.4092 | 0.1919 | 0.1841 | 0.1915 |  |
|  | **Overall** | 0.2236 | 0.2332 | 0.2241 |  |
